# Recommandation relative au dépistage de la chlamydia et de la gonorrhée en soins primaires chez les personnes non connues comme appartenant à un groupe à risque

**DOI:** 10.1503/cmaj.201967-f

**Published:** 2021-04-19

**Authors:** Ainsley Moore, Gregory Traversy, Donna L. Reynolds, John J. Riva, Guylène Thériault, Brenda J. Wilson, Melissa Subnath, Brett D. Thombs, pour le Groupe d’étude canadien sur les soins de santé préventifs

**Affiliations:** Département de médecine familiale (Moore), Université McMaster, Hamilton, Ont.; Agence de la santé publique du Canada ( Subnath, Traversy), Ottawa, Ont.; Département de médecine familiale et communautaire (Reynolds), Université de Toronto, Toronto, Ont.; Département de médecine familiale (Riva), Université McMaster, Hamilton, Ont.; Faculté de médecine (Thériault), Université McGill, Montréal, Qc; Division de santé communautaire et humanités (Wilson), Université Memorial, T.-N.–L.; Institut Lady Davis et Département de psychiatrie (Thombs), Hôpital général juif et Université McGill, Montréal, Qc.

**POINTS CLÉS**
- Les 2 infections bactériennes transmissibles sexuellement (ITS) les plus souvent rapportées au Canada, soit les infections à *Chlamydia trachomatis* et *Neisseria gonorrhoeae*, peuvent causer des complications telles que la maladie inflammatoire pelvienne, la douleur pelvienne chronique, la grossesse ectopique et l’infertilité.
- L’offre d’un dépistage opportuniste de la chlamydia en médecine de soins primaires pourrait réduire la maladie inflammatoire pelvienne chez les femmes, même si les données à ce sujet sont incertaines.
- La plupart des patients accordent probablement plus d’importance aux bénéfices potentiels du dépistage qu’aux préjudices qui peuvent y être associés; une faible proportion des personnes candidates admissibles au dépistage peut en subir des conséquences psychosociales négatives, telles que gêne, anxiété ou stigmatisation.
- Le Groupe d’étude canadien sur les soins de santé préventifs recommande un dépistage annuel de la chlamydia et de la gonorrhée chez les personnes de moins de 30 ans actives sexuellement lors de consultations en soins primaires, dans la mesure du possible (recommandation conditionnelle; données de très faible certitude).

Messages clés pour le public

- La chlamydia et la gonorrhée sont des infections transmissibles sexuellement communes qui répondent à l’antibiothérapie.
- Si vous avez moins de 30 ans et avez une vie sexuelle active, votre professionnel de la santé pourrait vous offrir un dépistage annuel de la chlamydia et de la gonorrhée, même si vous n’avez aucun symptôme.
- Le dépistage a pour but de découvrir et de traiter ces infections chez les personnes qui ne manifestent pas de symptômes, afin d’en prévenir les complications, comme la maladie inflammatoire pelvienne. Ces infections sont souvent asymptomatiques.
- Informez-vous auprès de votre médecin au sujet d’un dépistage plus fréquent qu’une fois par an si vous croyez être plus à risque (p. ex., vous avez déjà eu une infection transmissible sexuellement, vous avez eu des relations sexuelles non protégées, des relations avec plusieurs partenaires, ou avec différents partenaires, ou toute autre raison).
- La chlamydia et la gonorrhée sont des infections qui sont systématiquement signalées aux unités de santé publique locales. Cela facilite le traitement des personnes déclarées positives et permet d’informer leurs partenaires de façon confidentielle pour assurer le dépistage et le traitement si nécessaire.

Au Canada, la chlamydia et la gonorrhée (causées respectivement par les bactéries *Chlamydia trachomatis* et *Neisseria gonorrhoeae*) sont les infections bactériennes transmissibles sexuellement (ITS) les plus communes^1^,^2^, et le nombre de cas rapportés annuellement dans la population est en hausse depuis 2000^3^. En 2018, le nombre de cas rapportés pour l’année a été le plus élevé chez les personnes de 15–29 ans, avec des taux de 1,0 %–1,9 % pour la chlamydia et de 0,2 %–0,3 % pour la gonorrhée^4^, alors que les taux chez les plus de 30 ans étaient < 0,5 % pour la chlamydia et < 0,2 % pour la gonorrhée^4^. Par contre, beaucoup de personnes infectées sont asymptomatiques ou ne consultent pas et ne sont donc pas déclarées^3^. Si on tient compte de cette sous-déclaration, la prévalence réelle de la chlamydia chez les 15–29 ans pourrait atteindre 5 %–7 %^3^–^6^.

Chez les femmes, les conséquences d’une chlamydia non traitée sont notamment : cervicite chez 10 %–20 %^7^, maladie inflammatoire pelvienne chez 10 %–16 %^8^,^9^, infertilité chez jusqu’à 5 %^10^, douleur pelvienne chronique chez 3 %–8 %^10^,^11^ et grossesse ectopique chez jusqu’à 2 %^10^. Les conséquences de la gonorrhée peuvent inclure des taux de maladie inflammatoire pelvienne supérieurs à ceux que cause la chlamydia^12^. Chez les hommes, les conséquences de la chlamydia peuvent inclure l’épididymite chez jusqu’à 7 %, avec ou sans orchite^6^,^13^, et très rarement, l’infertilité^14^. Les conséquences de la chlamydia affectant les 2 sexes incluent l’urétrite chez 4 % des femmes et jusqu’à 3 % des hommes^15^, la pharyngite, la proctite, l’arthrite réactionnelle d’une durée de plus de 6 mois chez 1 %–4 % (en tenant compte de la chlamydia et de la gonorrhée)^16^,^17^ et l’infection gonococcique disséminée chez < 1 %, qui peut entraîner dans de rares cas la septicémie, la méningite, l’endocardite ou l’ostéomyélite^18^.

Le dépistage de la chlamydia et de la gonorrhée chez les personnes actives sexuellement pourrait réduire les complications cliniques et la transmission, mais doit être effectué uniquement si ses bénéfices excèdent les préjudices qui y sont associés^19^,^20^ et si l’utilisation des ressources est justifié.

Au Canada, le dépistage des ITS est le plus souvent offert de manière opportuniste par les médecins dans divers contextes de soins primaires (p. ex., médecine familiale, clinique de santé sexuelle, services de santé scolaires) lors de consultations liées ou non à des problèmes de santé sexuelle^21^. Le dépistage opportuniste diffère d’un programme systématique de dépistage dans la population, au cours duquel on envoie des invitations à se faire dépister à toutes les personnes admissibles, après quoi on évalue l’adoption du dépistage et ses résultats au moyen d’une structure provinciale centralisée.

La ligne directrice nationale de l’Agence de la santé publique du Canada préconise actuellement un dépistage de la chlamydia chez les femmes enceintes, un dépistage annuel chez les personnes de moins de 25 ans actives sexuellement et un dépistage ciblé chez les personnes à risque de plus de 25 ans (p. ex., ayant des antécédents d’ITS, ou travailleurs et travailleuses de l’industrie du sexe)^21^–^23^. Elle ne se fonde pas sur des revues systématiques formelles et n’inclut pas de recommandations pour le dépistage de la gonorrhée.

La dernière Ligne directrice sur le dépistage de la chlamydia du Groupe d’étude canadien sur l’examen médical préventif (désormais le Groupe d’étude canadien sur les soins de santé préventifs) remonte à 1996^24^; elle n’incluait pas non plus de recommandations concernant la gonorrhée. Le Groupe d’étude canadien a donc jugé nécessaire de mettre à jour la ligne directrice canadienne en tenant compte des données actuelles sur les bénéfices et préjudices potentiels du dépistage de la chlamydia et de la gonorrhée, et sur les valeurs et préférences des patients à cet égard.

## Portée

Cette ligne directrice s’adresse aux médecins de soins primaires qui sont en position d’offrir le dépistage opportuniste de la chlamydia et de la gonorrhée directement aux personnes actives sexuellement qui ne consultent pas spécifiquement pour une possible ITS et qui ne sont pas connus comme appartenant à un groupe à risque. Le lecteur devrait consulter les lignes directrices nationales, provinciales ou locales pertinentes pour le dépistage chez les personnes connues comme ayant des comportements à risque spécifiques (qui varient selon les régions, mais peuvent inclure les personnes qui ont de multiples partenaires sexuels, des antécédents d’ITS ou des rapports sexuels non protégés^22^,^25^,^26^). Ces mêmes lignes directrices sont utiles dans la prise en charge des personnes qui consultent pour des symptômes, les femmes enceintes, de même que pour la sélection des antibiotiques appropriés. Elles peuvent aussi servir de source d’information pour la notification aux partenaires, les tests de contrôle et les stratégies d’analyses médico-légales. Les termes « homme » et « femme » font référence au sexe (c.-à-d., attributs biologiques, et plus particulièrement, anatomie de l’appareil reproducteur ou sexuel à la naissance), à moins d’indication contraire.

## Recommandation

Nous recommandons un dépistage opportuniste annuel de la chlamydia et de la gonorrhée chez les personnes de moins de 30 ans actives sexuellement qui ne sont pas connues comme appartenant à un groupe à risque, lors de consultations en soins primaires, au moyen d’un autoprélèvement ou d’un prélèvement fait par le médecin (recommandation conditionnelle; données de très faible certitude).

Un sommaire de la recommandation est présenté dans l’encadré 1.

Encadré 1 : **Sommaire de la recommandation pour les médecins, les responsables des orientations politiques et les patients**

Nous recommandons un dépistage opportuniste annuel de la chlamydia et de la gonorrhée chez les personnes de moins de 30 ans actives sexuellement qui ne sont pas connues comme appartenant à un groupe à risque, lors de consultations en soins primaires, au moyen d’un autoprélèvement ou d’un prélèvement fait par le médecin (recommandation conditionnelle; données de très faible certitude).

Les professionnels devraient consulter les lignes directrices nationales, provinciales ou locales pertinentes pour le dépistage chez les personnes connues comme appartenant à des groupes à risque.

Dans la revue systématique réalisée pour la présente ligne directrice, toutes les études pertinentes (9 essais randomisés et contrôlés [ERC]^5^,^6^,^8^,^27^–^32^, 1 essai clinique contrôlé^33^ et 2 études de cohorte rétrospectives^34^,^35^) sur les bénéfices potentiels du dépistage de la chlamydia ont fourni des données indirectes (c.-à-d., de faible applicabilité) sur la façon d’offrir le dépistage et sur les possibles personnes candidates en soins primaires au Canada. Par exemple, 4 ERC ont offert le dépistage (indépendamment de son adoption) sur invitation, par la poste ou messages publics incitant au dépistage^6^,^29^, et non lors de discussions directes, et 1 ERC en grappe a appliqué des interventions en clinique (trousses incitant les médecins à offrir le dépistage)^5^ plutôt qu’un engagement direct des médecins, ce qui a généré une faible participation des médecins et peu d’offres de dépistage. Trois essais ont évalué uniquement les personnes ayant accepté le dépistage (acceptants du dépistage)^8^,^28^,^33^ et 1 essai a évalué une offre de dépistage chez des personnes présélectionnés pour leur intérêt à subir le dépistage (offre de dépistage, présélection)^31^; ces données sont indirectes en fonction des divers degrés d’intérêt et de l’adoption du dépistage chez les patients de soins primaires au Canada. Aucune étude sur les effets du dépistage de la gonorrhée sur les issues cliniques d’intérêt, n’a été recensée dans les populations présentant un risque général.

Onze études sur les préjudices associés au dépistage de la chlamydia ont été recensées. Un ERC a fait état des effets indésirables des antibiotiques^5^ et 10 études de cohortes non contrôlées ont fait état de préjudices psychosociaux^20^,^36^–^44^. Aucune étude ne s’est penchée sur les préjudices associés au dépistage de la gonorrhée.

Quatorze études ont examiné les valeurs et préférences relatives au dépistage de la chlamydia ou de la gonorrhée : 4 études ont calculé les mesures des valeurs des états de santé (mesure de la préférence à l’égard d’un état de santé particulier^45^) pour plusieurs bénéfices du dépistage de la chlamydia et de la gonorrhée^11^,^46^– ^48^, et 10 études (sondages et études qualitatives) se sont attardées à l’importance relative des bénéfices par rapport aux préjudices associés au dépistage de la chlamydia^49^–^58^.

### Bénéfices associés au dépistage

#### Offre de dépistage, indépendamment de son adoption

Une méta-analyse de 2 ERC (*n* = 141 362) a fait état de données de très faible certitude quant à une différence de minime à nulle pour ce qui est du taux de maladie inflammatoire pelvienne chez les femmes de 16–29 ans, sur une période de 1–3 ans avec un dépistage annuel de la chlamydia par autoprélèvements vaginaux (0,3 de plus sur 1000 [intervalle de confiance (IC) à 95 % de 7,6 de moins à 11 de plus])^5^,^6^.

Un ERC (*n* = 15 459) a fait état d’effets très incertains sur l’infertilité et de données de très faible certitude quant à une différence de minime à nulle pour ce qui est du taux de grossesses ectopiques chez les femmes de 21–24 ans, sur une période de 9 ans pour une offre unique de dépistage de la chlamydia par autoprélèvements vaginaux (0,2 de plus sur 1000 [IC à 95 % de 2,2 de moins à 3,9 de plus])^6^.

Une méta-analyse de 3 ERC (*n* = 41 709) a fait état de données de faible certitude quant à une différence de minime à nulle pour ce qui est de la transmission de la chlamydia chez les personnes de 15–29 ans, sur une période de 1–3 ans pour une offre unique de dépistage de la chlamydia par autoprélèvements vaginaux^5^,^27^ ou urinaires^24^,^26^ (5,4 de moins par 1000 [IC à 95 % de 21,0 de moins à 12,6 de plus])^27^,^29^.

#### Offre de dépistage, personnes présélectionnées intéressées au dépistage

Un ERC (*n* = 2607) — chez des femmes de 18–34 ans (81 % de moins de 24 ans) présélectionnées à l’aide d’un questionnaire sur le risque de chlamydia et ayant accepté une offre de rendez-vous en soins primaires (suggérant un intérêt pour le dépistage) — a fait état de données de faible certitude selon lesquelles offrir un dépistage unique de la chlamydia à l’aide d’un prélèvement au niveau cervical effectué par les médecins pourrait réduire la maladie inflammatoire pelvienne (15,4 de moins par 1000 [IC à 95 % de 3,0 à 21,3 de moins], nombre de personnes à dépister 65 [IC à 95 % de 47 à 333])^31^.

#### Acceptants du dépistage

Deux ERC et 1 essai clinique contrôlé (*n* = 30 652) ont fait état de données de faible certitude selon lesquelles les femmes de 15–29 ans ayant subi un dépistage unique de la chlamydia sur une période de 12–18 mois par autoprélèvements vaginaux^8^,^28^ ou urinaires^33^ pourraient avoir réduit leur risque de maladie inflammatoire pelvienne sur une période d’un an (5,7 de moins par 1000 [IC à 95 % de 10,8 de moins à 1,1 de plus])^8^,^28^,^33^.

### Préjudices associés au dépistage

Un ERC (*n* = 37 543 personnes testées; *n* = 4574 patients ayant reçu un diagnostic de chlamydia; nombre de personnes traités non précisé) n’a fait état d’aucun effet indésirable de l’antibiothérapie pour la chlamydia (données de très faible certitude)^5^. Des études de cohorte^20^,^36^–^44^ ont fait état de divers préjudices psychosociaux associés au dépistage; ils ont été synthétisés de façon narrative. Des données de faible ou très faible certitude ont indiqué que le dépistage pouvait susciter des sentiments de stigmatisation (p. ex., culpabilité, gêne, désapprobation sociale) ou d’anxiété reliée au risque d’infertilité, à la sexualité ou au risque d’infection pour une proportion petite à moyenne de personnes (50–400 par 1000 personnes soumises au dépistage). Le nombre de personnes affectées dans l’ensemble de la population admissible au dépistage est probablement moindre. La durée et la gravité exactes de ces symptômes sont inconnues.

### Valeurs et préférences des patients

Si l’on tient compte des bénéfices par rapport aux préjudices, les sondages et les études qualitatives ont révélé que les personnes qui ont envisagé le dépistage (*n* = 777)^49^–^55^ ou s’y sont soumises (*n* = 77)^56^–^58^ ont accordé une plus grande importance aux bénéfices pour la santé reproductive et la réduction de la transmission qu’au risque d’anxiété ou de stigmatisation lié au dépistage (données de très faible certitude). Aucune étude n’a tenu compte des valeurs des patients en lien avec les effets indésirables des médicaments.

De même, l’étude sur la participation des patients réalisée par le Groupe d’étude canadien pour cette ligne directrice (décrite à la section Méthodologie) a montré que les patients priorisent probablement les bénéfices du dépistage (tous jugés cruciaux ou importants) par rapport aux préjudices qui y sont associés (tous jugés importants) et préfèrent nettement avoir un dépistage, même après qu’on leur ait présenté les données sur le caractère incertain des données^59^,^60^.

En tenant compte de la priorisation relative des différents bénéfices associés au dépistage, les études ayant indiqué des valeurs des états de santé ont révélé que même si ces valeurs sont similaires d’un paramètre à l’autre pour les bénéfices^11^,^46^–^48^, lorsqu’on tient compte de la durée des états de santé, le fait d’éviter l’infertilité et la douleur pelvienne chronique aurait plus d’importance pour les femmes que l’évitement de la grossesse ectopique, de la maladie inflammatoire pelvienne ou de la cervicite (certitude faible à modérée)^61^.

### Utilisation des ressources

Le Groupe d’étude canadien n’a pas réalisé de revue systématique sur l’utilisation des ressources ou sur le rapport coût–efficacité. De l’avis du Groupe d’étude canadien, la recommandation de dépister tous les patients admissibles lors de consultations opportunistes pourrait entraîner des coûts additionnels (modérés) imputables au temps additionnel des cliniciens lors des visites et au prix des tests.

Les estimations du rapport coût–efficacité fondées sur des scénarios de dépistage opportuniste suggèrent que des taux de dépistage élevés plutôt que faibles pourraient améliorer le rapport coût–efficacité^62^ et que le dépistage pourrait avoir un bon rapport coût–efficacité au Canada étant donné que la probabilité de progression de la chlamydia vers la maladie inflammatoire pelvienne est d’au moins 10 %^63^, et ce, même si le degré de certitude est très faible.

### Faisabilité, acceptabilité, coûts et équité

Le dépistage fait actuellement partie de la pratique en soins primaires; il est donc jugé faisable et probablement acceptable pour les professionnels en soins primaires et les patients. À noter, 1 ERC a montré que 80 % des patients acceptaient le dépistage lorsqu’il leur était offert^64^ (même si le taux de dépistage global dans cet essai a été faible [24 %], car l’offre faisait défaut)^5^.

Le Groupe d’étude canadien prévoit que les parties prenantes de la santé publique et les autres responsables des orientations politiques trouveront la recommandation relative au dépistage acceptable, étant donné le nombre de personnes affectées, l’incidence croissante de la chlamydia et de la gonorrhée^4^ et l’existence d’un traitement efficace.

De l’avis du Groupe d’étude canadien, la recommandation améliorerait l’équité en matière de santé en normalisant le dépistage de routine chez les personnes actives sexuellement, réduisant de ce fait d’importants obstacles au dépistage, tels que la crainte de la désapprobation ou de la discrimination et le sentiment de stigmatisation^65^. De plus, comme les femmes assument la majeure partie des conséquences cliniques de l’infection, le dépistage chez les hommes (réservoir de l’infection pour les femmes) pourrait améliorer l’équité en matière de santé pour les femmes.

### Raison d’être

#### Bénéfices

La nature indirecte (faible applicabilité) des données disponibles pour orienter le dépistage opportuniste au Canada représente une source majeure d’incertitude, qui s’ajoute à la grande incertitude des données ou à leur absence en ce qui concerne certaines issues cliniques concernant le dépistage de la chlamydia et toutes les issues cliniques concernant le dépistage de la gonorrhée. Toutes les données sur les bénéfices étaient de faible ou très faible certitude, en partie en raison de leur caractère indirect et de leur imprécision (annexe 1, accessible en anglais au : www.cmaj.ca/lookup/doi/10.1503/cmaj.201967/tab-related-content). La maladie inflammatoire pelvienne pourrait diminuer chez les femmes qui acceptent et subissent le dépistage de la chlamydia^8^,^28^,^33^ ou qui s’y intéressent quand on le leur offre (faible certitude)^31^ (tableau 1). Des données de très faible certitude ont révélé une différence minime, voire nulle, pour ce qui est de la maladie inflammatoire pelvienne lorsque le dépistage de la chlamydia était offert, soit sur invitation par la poste, soit au moyen de trousses en clinique incitant au dépistage. Le Groupe d’étude canadien a jugé que les bénéfices réels d’un dépistage de la chlamydia offert directement par les professionnels en soins primaires canadiens qui sont en position d’identifier les personnes admissibles et de leur offrir le dépistage opportuniste se situeraient probablement dans la fourchette d’efficacité du dépistage qui a été observée (tableau 1).

**Tableau 1:** Effets du dépistage de la chlamydia sur la maladie inflammatoire pelvienne chez les femmes exposées à un risque général

Paramètre	Approche	N^bre^ et types d’études	Période de suivi, mois	Taux chez les femmes non soumises au dépistage	Taux chez les femmes soumises au dépistage (IC à 95 %)	Différence absolue (IC à 95 %)	Certitude des données probantes
Maladie inflammatoire pelvienne (MIP)	Offre de dépistage*, toutes les personnes admissibles	2 ERC^5^,^6^ *n* = 141 362	12–36	27,0 par 1000†	27,3 par 1000 (19,4 à 38,0)	0,30 de plus par 1000 (7,60 de moins à 11,0 de plus)	⊕⊖⊖⊖ TRÈS FAIBLE‡,§
	Offre de dépistage*, personnes sélectionnées	1 ERC^31^ *n* = 2607	12	27,0 par 1000†	11,6 par 1000 (5,70 à 24,0)	15,4 de plus par 1000 (3,00 à 21,30 de moins)	⊕⊕⊖⊖ FAIBLE§,¶
	Personnes qui acceptent	2 ERC, 1 ECC^8^,^28^,^33^ *n* = 30 652	12–18	27,0 par 1000†	21,3 par 1000 (16,2 à 28,1)	5,70 de moins par 1000 (10,8 de moins à 1,10 de plus)	⊕⊕⊖⊖ FAIBLE ‡,§

Remarque: CT = *Chlamydia trachomatis*, ECC = essai clinique contrôlé, ERC = essai randomisé et contrôlé, IC = intervalle de confiance, MIP = maladie inflammatoire pelvienne.

* Ces analyses représentent les résultats d’études qui ont examiné l’effet de l’offre du dépistage de la chlamydia ou de la gonorrhée à toutes les personnes admissibles, indépendamment du degré d’adoption. Une étude a utilisé une approche pour une offre de dépistage à une population présélectionnée de personnes intéressées au dépistage (offre de dépistage, personnes sélectionnées).

† Les effets sans dépistage présumaient qu’environ 6 % de la population féminine serait porteuse de la chlamydia (prévalence du risque général). Pour le paramètre MIP, on a présumé qu’environ 13 % des femmes porteuses de la chlamydia développeraient une MIP (0,78 % de la population totale) et qu’environ de 25 %–30 % des MIP de toutes causes sont attribuables à la chlamydia (MIP de toutes causes = 3,5 fois des suites de CT); 0,78 % × 3,5 = prévalence de 2,7 % de la MIP causée par la chlamydia dans le groupe non soumis au dépistage.

‡ Graves préoccupations en raison du caractère indirect.

§ Graves préoccupations en raison de l’imprécision.

¶ Graves préoccupations en raison du risque de biais.

La recommandation de soumettre au dépistage les personnes de moins de 30 ans se fonde sur le fait que la quasi-totalité des données provient d’études sur des personnes de ce groupe d’âge. De plus, les taux de chlamydia et de gonorrhée sont en hausse chez les personnes de 25–29 ans au Canada, avec des taux et des nombres totaux de cas similaires à ceux des 15–19 ans^4^,^60^. À l’inverse, les taux de chlamydia des 30–39 ans représentent moins de la moitié de ceux des 15–19 ans et des 24–29 ans, et moins du quart de ceux des 20–24 ans^4^. De même, les taux chez les 40–59 ans sont moins du quart de ceux des 30–39 ans^4^.

En tenant compte des particularités des réseaux sexuels, cette recommandation de soumettre aussi au dépistage les hommes actifs sexuellement vise à réduire la chlamydia et la gonorrhée et ses conséquences négatives pour les femmes, en raison de leur rôle dans la transmission de ces infections (même si on ne disposait d’aucune étude pour étayer cet argument).

Le Groupe d’étude canadien a formulé la recommandation de dépister la gonorrhée également (malgré l’absence de données à cet effet) étant donné que de nombreux cas de gonorrhée sont asymptomatiques^23^,^66^ et que jusqu’à 40 % des personnes atteintes de gonorrhée ont aussi la chlamydia^67^–^69^. La pratique canadienne actuelle dans les cliniques et les laboratoires est de jumeler les tests de dépistage de la gonorrhée et de la chlamydia sur un même spécimen (la plupart des tests d’amplification des acides nucléiques (TAAN) commerciaux servent à dépister les 2 agents pathogènes à la fois sur un même spécimen^25^). On ignore quels sont les coûts additionnels du dépistage simultané de la chlamydia et de la gonorrhée (par rapport, par exemple à la chlamydia seule), ils sont probablement minimes, étant donné que certains barèmes provinciaux incluent déjà un tarif unique pour le TAAN de la chlamydia et de la gonorrhée ensemble^70^.

#### Préjudices

Le Groupe d’étude canadien a accordé une priorité moindre aux données de très faible certitude quant à l’absence d’effets indésirables graves du traitement antibiotique pour la chlamydia et la gonorrhée et aux données incertaines quant aux préjudices psychosociaux associés au dépistage (anxiété, honte et stigmatisation) qu’éprouvera probablement une faible proportion des personnes admissibles au dépistage.

Le Groupe d’étude canadien a jugé que les bénéfices potentiels du dépistage de la chlamydia et de la gonorrhée visant à réduire la maladie inflammatoire pelvienne chez les femmes, malgré leur grande incertitude, l’emportent sur les préjudices possibles. Selon les données, la plupart des patients canadiens accordent plus d’importance aux bénéfices qu’aux préjudices associés au dépistage de la chlamydia et de la gonorrhée, même quand on leur soumet les données et le degré d’incertitude de celles-ci^59^,^60^. Donc, en tenant compte à la fois des bénéfices, des préjudices, et du caractère incertain des données, le Groupe d’étude canadien formule une recommandation conditionnelle en faveur du dépistage opportuniste de la chlamydia et de la gonorrhée en soins primaires chez les personnes de moins de 30 ans.

## Méthodologie

Le Groupe d’étude canadien sur les soins de santé préventifs est un comité indépendant de médecins et de méthodologistes qui formule des recommandations sur des interventions cliniques de prévention primaire et secondaire (https://canadiantaskforce.ca/?lang=fr). Un groupe de travail composé de 6 membres du Groupe d’étude canadien a rédigé cette recommandation avec le soutien scientifique du personnel de l’Agence de la santé publique du Canada.

Le groupe de travail a formulé ses questions de recherche et préparé le cadre analytique pour la revue systématique (annexe 2, accessible en anglais au : www.cmaj.ca/lookup/doi/10.1503/cmaj.201967/tab-related-content). Le Centre d’analyse et de synthèse des données probantes de l’Université de l’Alberta (Edmonton, Alberta) a procédé à la revue systématique sur laquelle se fonde la recommandation et a évalué l’efficacité du dépistage de la chlamydia et de la gonorrhée et l’importance relative pour les patients des issues cliniques (bénéfices et préjudices) associés au dépistage^61^.

Une interrogation a été réalisée à partir des bases de données Ovid MEDLINE, Ovid Embase, bibliothèque Wiley Cochrane, CINAHL via EbscoHost et Ovid PsycINFO depuis leur création et jusqu’au 26 janvier 2020 pour trouver les études admissibles, avec des interrogations additionnelles de la littérature grise. Pour l’importance relative des paramètres liés aux bénéfices potentiels, une revue de 2013 sur la mesure des valeurs des états de santé a aussi été mise à jour^71^. La revue systématique a été enregistrée (PROSPERO CRD42018100733) et un protocole de la revue a été publié^13^.

Dans la revue systématique, les bénéfices potentiels du dépistage analysés incluaient la baisse des cas de maladie inflammatoire pelvienne, de grossesse ectopique, de cervicite et de douleur pelvienne chronique chez les femmes; et chez les hommes et les femmes, la baisse de l’infertilité et de la transmission (prévalence). Les préjudices potentiels analysés incluaient les réactions indésirables graves aux antibiotiques et les conséquences psychosociales négatives du dépistage (p. ex., anxiété, détresse sexuelle, y compris violence conjugale, stigmatisation, blâme). Les seuils pour les différences minimalement importantes concernant les paramètres liés aux bénéfices ont été déterminés avant que le Groupe d’étude canadien n’examine les résultats. On trouvera plus de détails dans la revue systématique^13^,^61^.

Le Groupe d’étude canadien a utilisé l’approche GRADE (Grading of Recommandations, Assessment, Development and Evaluation) pour déterminer la certitude des données et la force de la recommandation (encadré 2). Voir annexe 3, accessible en anglais au : www.cmaj.ca/lookup/doi/10.1503/cmaj.201967/tab-related-content. La recommandation a été révisée et approuvée à l’unanimité par le Groupe d’étude canadien.

Encadré 2 : **Classification des recommandations**

Les recommandations sont classifiées selon le système GRADE (Grading of Recommandations Assessment, Development and Evaluation system)^72^. Qu’une recommandation soit forte ou conditionnelle dépend de facteurs tels que la certitude quant aux effets d’une intervention, y compris leur ampleur, de même que des estimations de la façon dont les patients évaluent et priorisent les paramètres d’intérêt mesurés, de la variabilité de ces estimations et d’une utilisation judicieuse des ressources.

**Fortes recommandations**

- Les fortes recommandations sont celles à propos desquelles le Groupe d’étude canadien sur les soins de santé préventifs considère que les effets escomptés d’une intervention en surpassent les conséquences indésirables (forte recommandation à l’appui de l’intervention) ou que les conséquences indésirables d’une intervention en surpassent les effets escomptés (forte recommandation à l’encontre de l’intervention). Une forte recommandation suppose que les intérêts de la plupart des personnes seront mieux servis par la mesure recommandée.
- Les fortes recommandations se fondent normalement sur des données probantes de grande certitude (c.-à-d., grande fiabilité de l’effet estimé d’une intervention). Les fortes recommandations peuvent préconiser une intervention (degré de confiance élevé quant aux bénéfices escomptés) ou la déconseiller (degré de confiance élevé quant à la probabilité de préjudices). Toutefois, il y a des circonstances où les recommandations fortes peuvent être envisagées en fonction de données de faible ou de très faible certitude ou en l’absence de données probantes^73^.
- En l’absence de données appuyant les bénéfices d’une nouvelle intervention préventive ou si pour conclure à ses possibles bénéfices il faut un fort degré de spéculation quant aux liens entre des données incertaines, et en présence d’une certitude élevée quant à des préjudices possibles pour certains patients ou quant à une utilisation indue de ressources précieuses, le Groupe d’étude canadien pourrait formuler une forte recommandation à l’encontre du déploiement de ladite intervention^74^. Cela concorde avec l’approche GRADE, selon laquelle de fortes recommandations sont parfois formulées à partir de données de faible certitude alliées à une certitude élevée concernant les préjudices ou l’utilisation des ressources, et en tenant compte de la valeur que le Groupe d’étude canadien accorde à l’utilisation judicieuse des ressources en soins primaires^74^.

**Recommandations conditionnelles**

- Les recommandations conditionnelles sont celles à propos desquelles les effets escomptés surpassent probablement les conséquences indésirables (recommandation conditionnelle en faveur d’une intervention) ou les conséquences indésirables surpassent probablement les effets escomptés (recommandation conditionnelle à l’encontre d’une intervention), mais une incertitude marquée existe. Les recommandations conditionnelles sont formulées lorsque la certitude des données probantes est plus faible, lorsque la frontière entre effets recherchés et conséquences indésirables est mince et lorsque le rapport dépend des valeurs et préférences des patients, ou lorsqu’il y a une forte variabilité entre les valeurs et les préférences des patients. Les cas où le rapport coût–bénéfice est ambigu, où les intervenants clés ne s’entendent pas sur l’acceptabilité ou la faisabilité de l’intervention, et où l’effet sur l’équité en matière de santé est indéterminé, conduiront probablement à une recommandation conditionnelle.
- Dans certains cas où on formule une recommandation conditionnelle concernant une intervention, les médecins sont encouragés à impliquer leurs patients dans un processus de prise de décision partagée, à reconnaître que différents choix seront appropriés selon les patients et à aider chacun d’entre eux à prendre une décision relative à une intervention qui est en accord avec ses valeurs et ses préférences.

Les données sont jugées de certitude élevée, modérée, faible ou très faible selon le degré de confiance du Groupe d’étude canadien à l’endroit de l’exactitude de l’estimation des effets.

Des renseignements supplémentaires sur la méthodologie utilisée par le Groupe d’étude canadien sont accessibles sur son site Web (https://canadiantaskforce.ca/methodologie/?lang=fr).

### Participation des patients

L’équipe chargée de l’application des connaissances à l’Hôpital St. Michael de Toronto a recruté des membres du public de partout au Canada, par le biais de publicités dans des sites Web publics (p. ex., Craigslist et Kijiji) au nom du Groupe d’étude canadien pour participer à 2 étapes de la préparation de la ligne directrice. Lors d’une première étape, 16 participants actifs sexuellement (9 identifiés comme femmes et 7 comme hommes) âgés de 24–38 ans ont classé l’importance des issues cliniques liés au dépistage dans un sondage en ligne et ont participé à un groupe de discussion pour présenter leurs arguments et discuter des facteurs qui influent sur l’importance qu’ils accordent aux diverses issues cliniques^59^,^75^. Les issues cliniques jugées cruciales et importantes dans le processus décisionnel par les participants et les membres du Groupe d’étude canadien ont été inclus dans la revue systématique. Après l’examen des données, 17 participants de 24–38 ans actifs sexuellement (13 identifiés comme hommes, 3 comme femmes et 1 comme non-binaire) ont classé l’importance des paramètres liés au dépistage dans un sondage en ligne, cette fois après qu’on leur ait remis un sommaire des résultats de la revue systématique^60^.

Des outils de transfert des connaissances orientés par les commentaires des médecins et des patients ont été mis au point pour appuyer l’application de la recommandation (accessible au https://canadiantaskforce.ca/tools-resources/chlamydia-et-gonorrhee/?lang=fr).

### Revue externe et par des experts de contenu

Le protocole^13^, la revue systématique^61^ et l’ébauche de la ligne directrice ont été révisés par des parties prenantes, des pairs-réviseurs, des experts cliniques et des experts de contenu (voir Remerciements). Les experts cliniques et les experts de contenu ont agi à titre de consultants auprès du groupe de travail; ils ont participé aux réunions du groupe de travail et passé en revue les documents pour en vérifier l’exactitude, mais ils n’ont exercé aucune influence et n’ont eu aucun droit de regard sur l’orientation de la recommandation ou la détermination de sa force.

### Gestion des intérêts concurrents

Le Groupe d’étude canadien observe les principes du Guidelines International Network pour la divulgation des intérêts concurrents et leur gestion^76^,^77^. Le comité de supervision du Groupe d’étude canadien chargé d’évaluer les intérêts concurrents et de statuer sur ces derniers se compose du président et de la vice-présidente du Groupe d’étude canadien et de la directrice de la Division des lignes directrices et de la santé mondiale de l’Agence de la santé publique du Canada^77^.

Le soutien financier du Groupe d’étude canadien sur les soins de santé préventifs provient de l’Agence de la santé publique du Canada. Les vues de l’organisme subventionnaire n’ont aucunement influé sur le contenu de la recommandation.

Tous les membres du Groupe d’étude canadien doivent déclarer leurs intérêts financiers ou autres intérêts pertinents annuellement lorsque de nouveaux thèmes sont choisis et à chaque rencontre en personne du Groupe d’étude canadien (3 fois l’an). Les énoncés de conflits d’intérêts sont accessibles au public dans le site Web du Groupe d’étude canadien. Aucun conflit d’intérêts n’a été déclaré par les membres du Groupe d’étude canadien pour cette ligne directrice.

Les experts cliniques et les experts de contenu dévoilent aussi tout intérêt pertinent au début de leur participation et annuellement par la suite (voir annexe 4, accessible en anglais au : www.cmaj.ca/lookup/doi/10.1503/cmaj.201967/tab-related-content). Le Groupe d’étude canadien a jugé qu’aucun des intérêts concurrents ne représentait un conflit d’intérêts pouvant empêcher la participation de quelconque expert clinique ou expert de contenu.

## Mise en œuvre

Pour mettre en œuvre cette recommandation sur le dépistage, on conseille aux médecins en soins primaires d’identifier les personnes admissibles (actives sexuellement, âgées de moins de 30 ans) ne consultant pas pour une possible ITS, et de leur offrir le dépistage opportuniste de la chlamydia et de la gonorrhée (c.-à-d., sans une consultation distincte pour le dépistage, et non seulement lors de consultations relatives à la santé sexuelle). Comme on le mentionne plus haut, les résultats d’un ERC suggèrent que l’acceptation du dépistage par les patients est élevée quand il leur est offert^64^. Comme les personnes à haut risque de chlamydia et de gonorrhée ne s’identifient pas comme telles spontanément ou ne sont pas facilement identifiées par les médecins, offrir le dépistage d’emblée, s’applique à tous les personnes actives sexuellement sans que le médecin connaisse leur appartenance à un groupe à risque. Les infections transmissibles sexuellement sont associées à des sentiments de honte et de gêne et à une stigmatisation substantielle, qui pourraient empêcher les patients de consulter pour dépistage et traitement^65^,^78^. Offrir le dépistage de routine à toutes les personnes actives sexuellement serait une façon de réduire la stigmatisation associée aux tests pour les ITS^78^. Les personnes qui veulent subir un dépistage et qui sont connues comme appartenant à un groupe à risque devraient être traitées conformément aux lignes directrices nationales, provinciales ou locales pertinentes qui s’appliquent à ces populations. L’activité sexuelle peut généralement être définie par des rapports oraux, vaginaux ou anaux.

Le consentement éclairé, qui est requis pour procéder au dépistage des ITS, est une autre dimension de son application. Les principaux enjeux à considérer sont la protection des renseignements personnels, le signalement des résultats positifs aux autorités de santé publique locales et la notification aux partenaires, le cas échéant. Le dépistage des infections transmissibles sexuellement peut susciter des sentiments de gêne et d’anxiété chez certains patients. L’offre du dépistage requiert une sensibilité à la stigmatisation et à la crainte de la désapprobation sociale, surtout au regard des questions de genre, de culture, de comportement et autres.

Même si on ignore quel est l’intervalle optimal entre les dépistages, une offre annuelle de dépistage serait appropriée pour les personnes exposées à un risque général, tout en reconnaissant que les consultations en soins primaires pourraient être moins fréquentes. La plupart des études recensées ont utilisé le dépistage annuel^61^ et dans 1 étude, la majorité des cas de maladie inflammatoire pelvienne survenant dans l’année affectaient des personnes qui étaient chlamydia-négatives au départ (risque général)^8^. Un résultat faux positif pourrait occasionner des préjudices sans avoir d’effets positifs pour certaines personnes, particulièrement là où la prévalence de la chlamydia dans la population est moins élevée (p. ex., 2 %–3 %) car le nombre de faux positifs par TAAN peut être assez élevé (p. ex., environ 30 %–60 % avec des spécificités de 97 %–99 %).

Même si nous n’avons pas relevé de données susceptibles d’orienter spécifiquement les stratégies de dépistage, son acceptabilité et son adoption^79^–^81^ seraient meilleures avec des modes de prélèvement minimalement invasifs comme les autoprélèvements vaginaux chez les femmes et les spécimens d’urine chez les hommes, ces 2 types de prélèvements étant plus précis (TAAN)^82^. Les prélèvements effectués par les médecins sont probablement acceptables et faisables lors de certaines consultations (p. ex., test de Pap)^83^. Au bout du compte, la préférence des patients et le scénario clinique orienteront probablement la méthode de prélèvement. On rappelle aux médecins d’envisager des prélèvements pharyngés et rectaux s’ils sont indiqués au plan clinique, même si nous n’avons relevé aucune donnée sur leur pertinence.

Pour les cas d’abus réels ou présumés chez des enfants, on oriente les médecins vers les autorités locales, provinciales et territoriales (santé publique, protection de la jeunesse, pédiatres et experts cliniques) pour le dépistage, le traitement, le signalement et la prise en charge des ITS.

## Suivi et évaluation

Les taux d’offre et d’adoption du dépistage chez les patients en soins primaires sont une mesure de rendement clé pour cette ligne directrice. Les taux de chlamydia et de gonorrhée rapportés en sont une autre.

## Autres lignes directrices

Les lignes directrices d’autres groupes canadiens^22^,^25^,^26^ et étrangers^84^– ^86^ recommandent aussi aux médecins d’offrir le dépistage opportuniste de la chlamydia aux personnes actives sexuellement. Certaines recommandent aussi le dépistage de la gonorrhée (tableau 2). La présente recommandation fixe la limite d’âge à 29 ans (voir la raison d’être de la recommandation ci-dessus)^4^, tandis que d’autres lignes directrices la fixent à 25 ans, sauf l’Australie, qui la fixe à 30 ans.

**Tableau 2:** Lignes directrices canadiennes (nationales et provinciales) et internationales sur le dépistage de la chlamydia et de la gonorrhée

Organisation	Recommandation
Groupe d’étude canadien sur les soins de santé préventifs (ligne directrice actuelle, 2021)	Nous recommandons un dépistage opportuniste annuel de la chlamydia et de la gonorrhée chez les personnes de moins de 30 ans actives sexuellement qui ne sont pas connues comme appartenant à un groupe à risque, lors de consultations en soins primaires, au moyen d’un autoprélèvement ou d’un prélèvement fait par le médecin (recommandation conditionnelle; données de très faible certitude).
Agence de la santé publique du Canada (2020)^22^	**Chlamydia** Le dépistage de *Chlamydia trachomatis* est recommandé chez quiconque présente des facteurs de risque à l’égard de cette infection. Recommandations pour le dépistage de *C. trachomatis*: Dépistage annuel: Âge < 25 ans Hommes gais, bisexuels et autres qui ont des relations sexuelles avec des hommes ou des personnes transgenres Dépistage ciblé: Offrir le dépistage et effectuer des contrôles selon les facteurs de risque chez les personnes ≥ 25 ans
Santé publique Ontario (2018)^25^	**Gonorrhée** Offrir le dépistage aux personnes actives sexuellement asymptomatiques qui ont des facteurs de risque à l’égard de la gonorrhée. En Ontario, les facteurs de risque qui revêtent une importance particulière chez les personnes ayant des rapports sexuels non protégés incluent: Femmes actives sexuellement de moins de 25 ans Hommes actifs sexuellement qui ont des relations sexuelles avec des hommes Autres facteurs de risque énumérés dans les *Ligne directrices canadiennes sur les infections transmissibles sexuellement*^23^ Lors d’un dépistage simultané de la gonorrhée et de la chlamydia: TAAN urinaire chez les hommes TAAN vaginal (1^er^ choix) ou urinaire (2^e^ choix) chez les femmes si on ne procède pas à un examen gynécologique TAAN cervical ou vaginal (1^er^ choix) ou urinaire (2^e^ choix) chez les femmes si on procède à un examen gynécologique
Ministère de la santé et des Services sociaux du Québec (2019)^26^	**Chlamydia** Dépistage au moins 1 fois l’an recommandé: Hommes et femmes de 25 ans et moins sexuellement actifs sans autres facteurs de risque Hommes et femmes ayant de nouveaux partenaires sexuels ou ayant eu > 1 partenaire en même temps depuis le plus récent test Personnes ayant eu 1 partenaire anonyme ou > 3 partenaires sexuels au cours de la dernière année Hommes ayant des relations sexuelles avec des hommes Travailleurs et travailleuses de l’industrie du sexe et leur clientèle (Dans certains cas) Personnes provenant d’une région où les infections transmissibles sexuellement et par le sang sont endémiques **Gonorrhée** Dépistage au moins 1 fois l’an recommandé: Hommes (selon la région) et toutes les femmes de 25 ans et moins actives sexuellement sans autres facteurs de risque Femmes ayant de nouveaux partenaires sexuels ou ayant eu > 1 partenaire en même temps depuis le plus récent test Personnes ayant eu 1 partenaire anonyme ou > 3 partenaires sexuels au cours de la dernière année Hommes ayant des relations sexuelles avec des hommes Travailleurs et travailleuses de l’industrie du sexe et leur clientèle (Dans certains cas) Personnes provenant d’une région où les infections transmissibles sexuellement et par le sang sont endémiques
US Preventive Services Task Force (USPSTF) (2014)^84^	**Femmes actives sexuellement** Le USPSTF recommande le dépistage de la chlamydia chez les femmes de 24 ans et moins actives sexuellement et chez les femmes plus âgées exposées à un risque accru d’infection (*recommandation de grade B*). **Hommes actifs sexuellement** Le USPSTF conclut que les données actuelles sont insuffiisantes pour évaluer le rapport entre les bénéfices et les préjudices associés au dépistage de la chlamydia et de la gonorrhée chez les hommes (*énoncé I*).
Public Health England (2018)^85^	**Chlamydia** Annuellement ou lors d’un changement de partenaire sexuel, offrir les tests aux hommes et aux femmes de moins de 25 ans s’ils ont déjà eu des rapports sexuels. Les professionnels devraient profiter de chaque occasion pour proposer le dépistage de la chlamydia en soins primaires et l’accès à des services de santé sexuelle, génésique et génito-urinaire.
Australasian Sexual Health Alliance (2018)^86^	**Test pour la chlamydia dans les situations suivantes:** Âge < 30 ans et actif sexuellement Changement de partenaire au cours des 12 derniers mois ITS au cours des 12 derniers mois Partenaire sexuel ayant eu une ITS Risque accru de complications d’une ITS; p. ex., interruption de grossesse ou pose de stérilet Signes ou symptômes potentiels de chlamydia Demande formulée par les patients lors d’une consultation en santé sexuelle

Remarque: ITS = infection transmissible sexuellement, TAAN = test d’amplification des acides nucléiques, USPSTF = United States Preventive Services Task Force.

## Lacunes dans les connaissances

Nous n’avons recensé aucun essai ayant effectué le dépistage opportuniste de la chlamydia ou de la gonorrhée de la façon dont il serait offert en soins primaires au Canada. Nous disposions en outre de données limitées sur les résultats du dépistage de la chlamydia ou de la gonorrhée sur la santé des hommes ou de leurs partenaires féminines spécifiquement (compte tenu des réseaux sexuels). Pour ainsi dire aucune étude n’a inclus de personnes de plus de 30 ans (peut-être en raison de la faible prévalence dans cette population). Des études devraient comparer les effets sur la santé de différents intervalles ou de différentes stratégies de dépistage en soins primaires.

## Limites de l’étude

Des seuils de différence minimalement importante ont été établis en ce qui concerne les issues cliniques en lien avec les bénéfices pour déterminer l’ampleur des effets et la certitude des données. Ces seuils ont été établis par les médecins du Groupe d’étude canadien et des experts de contenu en tenant compte des données épidémiologiques et de l’histoire naturelle canadiennes^13^,^61^. Nous n’avons pas sollicité les commentaires des patients au sujet de ces seuils, ce qui serait utile pour connaître leur point de vue sur certaines interventions préventives comportant des préjudices substantiels.

Nous n’avons pas mesuré les effets du traitement de la gonorrhée sur la résistance antimicrobienne étant donné que nous nous intéressions davantage aux paramètres importants pour les patients. Il ne s’agit pas nécessairement d’une limite, mais le Groupe d’étude canadien a fait des choix *a priori* de regrouper les données issues des études d’une façon qui peut être différente d’autres groupes. Nous n’avons pas mesuré l’effet moyen du dépistage parmi tous les essais retenus en raison de notre intérêt préalable pour les effets de l’offre de dépistage indépendamment de son adoption, et en raison de l’importante variabilité des protocoles des études à cet égard. Les responsables des orientations politiques moins intéressés par cette variabilité pourraient analyser différemment les données et l’ampleur des effets, sans que cela modifie l’orientation de la recommandation.

## Conclusion

Le dépistage opportuniste de la chlamydia et de la gonorrhée chez les personnes de moins de 30 ans actives sexuellement confère des bénéfices incertains, mais potentiellement importants, particulièrement pour la prévention de la maladie inflammatoire pelvienne chez les femmes. On prévoit des préjudices psychosociaux relativement bénins et les patients accordent probablement plus d’importance aux bénéfices potentiels du dépistage qu’aux préjudices qui y sont associés. Le Groupe d’étude canadien formule une recommandation conditionnelle en faveur du dépistage lors des consultations en soins primaires, chez les personnes de moins de 30 ans actives sexuellement non connues comme appartenant à un groupe à risque à l’égard de la chlamydia et de la gonorrhée. Un consentement éclairé doit être obtenu avant de procéder au dépistage.
